# The multisystem impact of perimenopausal hormonal fluctuations on chronic disease risk

**DOI:** 10.3389/fendo.2026.1894588

**Published:** 2026-08-10

**Authors:** Lijia Luo, Lei Li, Xi Wang, Wenpei Bai

**Affiliations:** 1 School of Basic Medical Sciences, Capital Medical University, Beijing, China; 2 National Key Laboratory of Intelligent Tracking and Forecasting for Infectious Diseases, Beijing Ditan Hospital, Capital Medical University, Beijing, China; 3 Beijing Institute of Infectious Diseases, Beijing, China; 4 Beijing Key Laboratory of Viral Infectious Diseases, Beijing Ditan Hospital, Capital Medical University, Beijing, China; 5 National Center for Infectious Diseases, Beijing Ditan Hospital, Capital Medical University, Beijing, China; 6 Department of Oncology, Capital Medical University, Beijing, China; 7 Beijing Shijitan Hospital, Capital Medical University, Beijing, China

**Keywords:** aging, estrogen, immunity, menopause, women

## Abstract

Hormonal shifts during menopause are crucial to women’s health, characterized by a reduction in estrogen levels and an elevation in circulating androgen, which may result from either natural or surgical menopause. This review synthesizes current knowledge on the multifaceted mechanisms by which menopausal hormonal fluctuations reshape systemic health span. This review synthesizes current knowledge of the complex mechanisms between hormonal changes and immune cell interactions, highlighting how these relationships can influence disease risk during the menopausal transition. We also emphasize how these neuroendocrine-immune-microbial disruptions facilitate the pathogenesis of neurodegenerative disorders, cardiovascular diseases, osteoporosis and the evolution of tumorigenic microenvironments. Finally, we discuss key challenges and future research directions in related research with goal of informing novel strategies to prevent and treat menopause-related diseases.

## 1 Introduction

**Figure 1:**
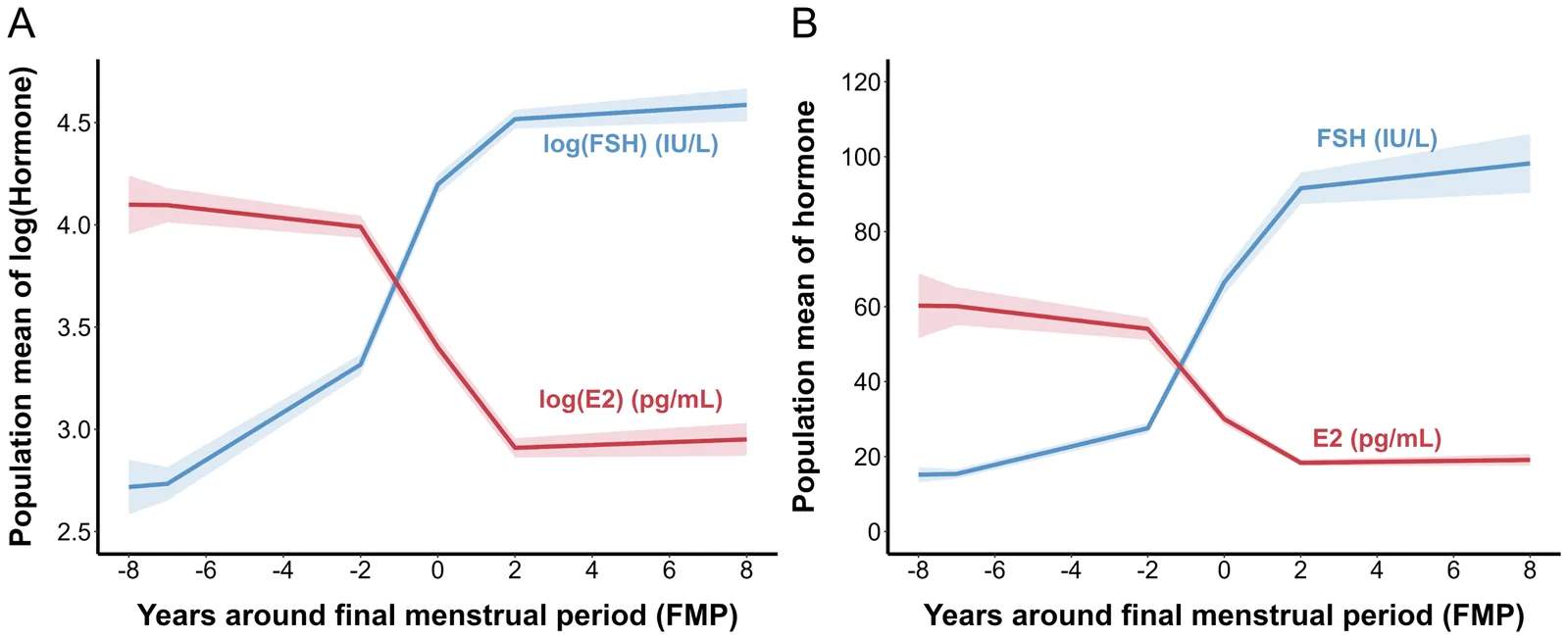
Trends in serum follicle-stimulating hormone (FSH) and estradiol (E2) levels around the final menstrual period. **(A)** Adjusted population means (95% CI) for natural logarithm-transformed FSH and E2 across years around the final menstrual period. **(B)** Back-transformed FSH and E2 levels across years around the final menstrual period. In both A and B, FSH levels increase as the FMP approaches, whereas E2 levels decline, with the most marked changes occurring in the period surrounding the FMP. The x-axis represents years before and after the final menstrual period. The y-axis is unitless for **(A, B)**. The units of hormone are marked in the corresponding curves. Dashed lines indicate 95% CI. FSH, follicle-stimulating hormone; E2, estradiol; FMP, final menstrual period; CI, confidence interval. Redrawn from Randolph JF Jr et al. (2011) (6) with permission from the copyright holder.

Menopause is the ultimate termination of ovarian function, characterized by reproductive hormone production decline and fertility loss. The average age of menopause is approximately 49 (1). Hormonal changes during the menopause transition result in menopausal symptoms, mainly comprising vasomotor symptoms (VMS), such as hot flushes and night sweats, urogenital changes, disordered sleep, and negative mood (1). A comprehensive search revealed that the prevalence of menopausal symptoms varies. The most prevalent symptoms include joint and muscular discomfort (65.43%, 95% confidence interval [CI] 62.51–68.29), while the least prevalent symptom is formication (20.5%, 95% CI 13.44–28.60) (2). For the past century, estrogens have been utilized in treatments designed to alleviate these effects, known as menopausal hormone therapies and sex hormone replacement therapies (HRT). Understanding the regulatory mechanisms of hormonal changes is essential for preventing and treating the menopausal effects. The dynamic changes in circulating follicle-stimulating hormone and estradiol (E2) levels during the menopausal transition are illustrated in **Figure 1**.

During the menopausal transition, drastic changes in the concentrations of sex steroids (including estrogens, androgens, and progestins) affect the innate and adaptive immune responses which impair the stimulation of immune-active cells, disrupting metabolic balance and contributing to the manifestation of menopausal symptoms, potentially increasing susceptibility to infections and autoimmune conditions (3). Additionally, they affect the microbiome and gut health, which are critical for immune system regulation (4). Older females exhibit a higher prevalence of inflammatory intermediate and non-classical monocytes compared to younger individuals. These specific monocyte subsets are the most inflammatory when examined ex vivo, and their frequency is linked to indicators of inflammaging (5).

The concentrations of sex steroids and signaling modulates immune responses primarily by interacting with estrogen receptors (ER), which regulate gene expression related to cytokine production and cell signaling. ER signaling can transcriptionally regulate immune cell function and dampers inflammation in part through effects of neutrophils. Furthermore, estrogen modulates the activation and functions of macrophages and other innate immune cells (3).

Surgical menopause is the sudden and complete termination of ovarian function, resulting in menopause symptoms, which can occur in the early postoperative period and can be severe. HRT can ease these symptoms; however, it does not entirely resolve them. Women who have received bilateral salpingo-oophorectomy(BSO) are recommended to be given HRT at least until they reach the natural age of menopause (7). HRT is an effective treatment option that can improve menopausal symptoms, such as VMS, genitourinary syndrome, and chronic disorders. However, a large-scale Women’s Health Initiative (WHI) trial demonstrated that HRT increases the risk of stroke and venous thromboembolic disease (8).

Hormonal changes during menopause lead to alterations in signaling pathways, triggering numerous menopausal symptoms and influencing the incidence and severity of certain diseases. Although HRT provides relief for some menopausal and disease-related symptoms, its application in disease management remains controversial. For example, there are debates surrounding the cardiovascular protective effects of HRT, its use in autoimmune patients, and its impact on Alzheimer’s disease (AD) and Parkinson’s disease (PD). Furthermore, the long-term efficacy and safety of HRT require extended research to establish global applicability.

In this review, we primarily address hormonal shifts around menopause and their impact on cardiovascular, autoimmune, neurological, neoplastic diseases and osteoporosis before and after menopause, including the influence of HRT on these changes.

## 2 Estrogen and immune dysregulation across the menopausal transition

**Figure 2:**
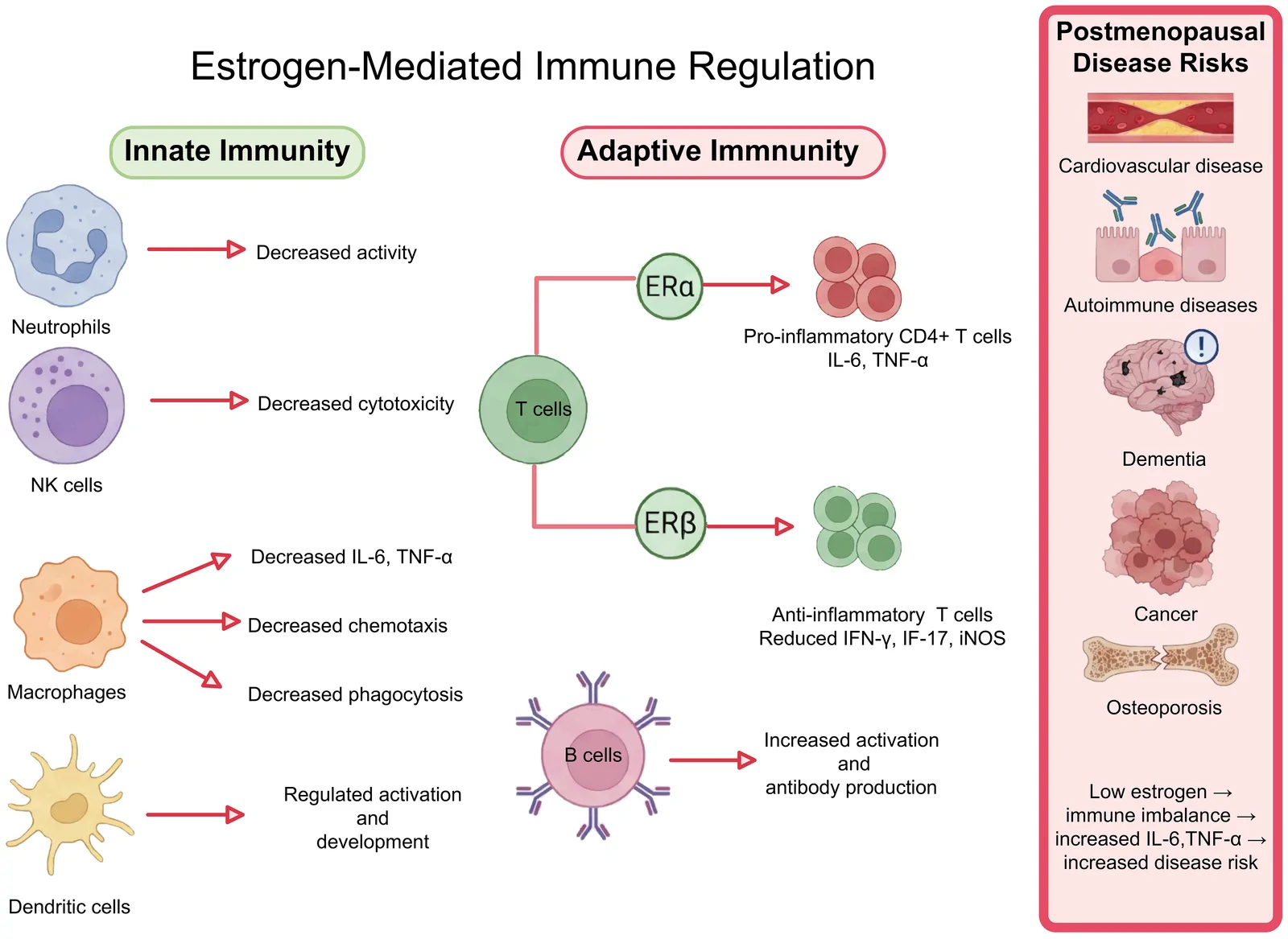
Estrogen-mediated immune regulation and its shift during menopause. Estrogen signaling exerts broad immunomodulatory effects across innate and adaptive immune cells. In innate immunity, estrogen decreases neutrophil activity, reduces NK cell cytotoxicity, and attenuates macrophage function, including cytokine production (IL-6, TNF-α), phagocytosis, and chemotaxis. It also promotes DC activation and development. In adaptive immunity, estrogen promotes anti-inflammatory T-cell polarization while restraining pro-inflammatory CD4^+^ T-cell responses. These effects are mediated through estrogen receptors: ERα signaling is associated with pro-inflammatory CD4^+^ T-cell responses, whereas ERβ signaling promotes anti-inflammatory T-cell phenotypes, characterized by reduced production of IFN-γ, IL-17, and iNOS. Estrogen also enhances B-cell activation and antibody production. Higher estrogen levels favor anti-inflammatory immune responses. However, during menopause, declining estrogen levels shift immune balance toward a pro-inflammatory state, with increased levels of cytokines such as IL-6 and TNF-α and a predominance of pro-inflammatory immune cells. This immune imbalance increases the risk of cardiovascular diseases, autoimmune diseases, dementia, cancer, and osteoporosis, as detailed in the article. ERα, estrogen receptor alpha; ERβ, estrogen receptor beta; NK, natural killer cell; DC, dendritic cell; IL-6, interleukin-6; TNF-α, tumor necrosis factor-alpha; IFN-γ, interferon-gamma; IL-17, interleukin-17; iNOS, inducible nitric oxide synthase. Created by the authors based on the findings summarized in Refs (17–126).

Estrogen signaling plays a central role in immune regulation by directly limiting inflammatory processes through multiple cellular and molecular mechanisms during the menopause transition and menopause period. The major immunomodulatory effects of estrogen on innate and adaptive immune cells during menopause are summarized in **Figure 2**. These include modulation of neutrophil activity and suppression of natural killer (NK) cell function, attenuation of macrophage cytokine production, phagocytosis, and chemotaxis, regulation of dendritic-cell development and activation, and promotion of anti-inflammatory T-cell polarization while restraining proinflammatory T subsets (3). Women with natural and surgical menopause exhibit high levels of inflammatory cytokines and a polarization toward pro-inflammatory immune cells, suggesting that loss of estrogen plays an important role in the development of inflammation (9, 10). Low estrogen levels are pro-inflammatory and promote pro-inflammatory T-cells, whereas high estrogen levels suppress inflammation and stimulate anti-inflammatory T-cells (11).

Studies have shown that menopausal women exhibit increased levels of pro-inflammatory cytokines, such as interleukin-6 (IL-6) and tumor necrosis factor-alpha (TNF-α), signaling a shift toward a more inflammatory immune profile. This pro-inflammatory state can lead to the development of chronic diseases, including cardiovascular disease, which are more prevalent in postmenopausal women (11).

Exogenous administration of 17β-estradiol (E2) has been found to reverse elevated inflammatory cytokine levels in ovariectomized imiquimod-induced psoriatic inflammatory mice. A similar phenomenon has been observed in esr1f/fesr2f/−flysm-Cre+ mice, in which ERs are specifically knocked out in neutrophils and macrophages (12). E2 also mediates dendritic cell (DC) development and activation through estrogen receptor alpha (ERα). In a murine model of experimental autoimmune encephalomyelitis (EAE), ERα deficiency in DCs increases CD4+ effector T cells, resulting in greater neurodegeneration (3). This demonstrated the importance of estrogen (mostly in the form of E2) regulation role.

In contrast, ER signaling exerts cell-specific effects in T cells. In autoimmune disease mouse models, ERα signaling in CD4+ T cells is pro-inflammatory, whereas Estrogen receptor beta (ERβ) signaling is anti-inflammatory (13, 14). In both mice and humans, ERβ signaling in T cells tends to suppress inflammatory T-cell responses. In the EAE model, ERβ signaling reduces CD4+ and CD8+ T cell aggregation in the central nervous system (CNS), as well as interferon-gamma (IFN-γ), interleukin-17, and inducible nitric oxide synthase (iNOS) production and disease severity (12). T-cell ERβ expression has been found to be lower in patients with multiple sclerosis or severe systemic lupus erythematosus (SLE) than in healthy controls (3).

Estrogen generally promotes the activation state of B cells by affecting the expression of necessary surface receptors and the intracellular signaling pathways required for their proliferation, differentiation, and activation (3, 15, 16). Furthermore, as estrogen regulates antibody production, females exhibit stronger antibody responses than males (15).

## 3 Progesterone’s immunomodulatory role in immune homeostasis

Progesterone has a significant impact on immune function during menopause as it possesses immunomodulatory properties that help maintain immune homeostasis. It can regulate the activity of various immune cells, including T cells, B cells, and macrophages. Specifically, progesterone promotes the differentiation of T helper 2 (Th2) cells, which are associated with anti-inflammatory responses. This shift helps reduce the likelihood of excessive inflammation, which can be detrimental to postmenopausal women (3).

Progesterone can modulate cytokine profiles, enhancing the production of anti-inflammatory cytokines such as interleukin-4 (IL-4) and interleukin-10 (IL-10) while suppressing pro-inflammatory cytokines such as IL-6 and TNF-α. This balance is crucial for maintaining immune tolerance and preventing autoimmune responses (3).

A relative decrease in progesterone production occurs during the luteal phase. Progesterone generally suppresses immune cell function. Studies employing mouse models have shown that the inhibitory effects of progesterone are mediated by the suppression of nuclear factor kappa-light-chain-enhancer of activated B cells (NF-κB) activation in innate immune cells, thereby reducing cyclooxygenase-2 (COX-2) enzymatic activity and the synthesis of pro-inflammatory cytokines, such as tumor necrosis factor (TNF) and interleukin-12 (IL-12) (127). However, most studies have focused on the responses during pregnancy.

## 4 Cortisol in immune regulation during the menopausal transition

Cortisol levels progressively increase from the early menopausal transition period to the late menopausal period and then decrease by the early postmenopausal period. The free androgen index, calculated as total testosterone divided by the sex hormone-binding globulin (SHBG) levels, increases over time following the final menstrual period, which is directly caused by the significant decrease in SHBG levels (128).

Testosterone can be enzymatically aromatized into estrogens that signal through the ERs or converted into other androgens, such as dihydrotestosterone, which signal through the androgen receptor (AR) (129).

AR signaling promotes the number of neutrophils *in vivo*, as evidenced in mouse model studies showing that the absence of either androgens or AR signaling results in reduced circulating neutrophil counts (130). In mouse models of experimental autoimmune myocarditis, AR suppresses the increase in anti-inflammatory macrophages and cytokine production, and this suppression is associated with reduced signal transducer and activator of transcription 3 (STAT3)/suppressor of cytokine signaling 3 (SOCS3) signaling, leading to inflammation during acute disease (131). However, the immunomodulatory effects of AR are context-dependent, as AR signaling can exert divergent or even opposing roles in inflammatory diseases such as allergic asthma and lung inflammation (130).

AR signaling directly and indirectly dampens T-cell-mediated inflammation by suppressing the activation and function of T cells to promote tolerance (130). In both mouse models and humans, AR activity has been shown to suppress CD8^+^ T cell function and contribute to the exhaustion or reduced proliferation of these cells, highlighting a mechanistic basis for sex differences in adaptive immunity (132). Furthermore, AR signaling may also suppresses B cell activity, with implications for autoimmunity, as AR-mediated suppression of adaptive immune responses affects both T and B lymphocyte compartments (130).

## 5 Menopause-related cardiovascular disease

### 5.1 Clinical significance of menopause-related hormonal changes in cardiovascular disease

The cardioprotective effects of endogenous estrogens delay the incidence of ischemic heart disease and myocardial infarction in women by approximately a decade compared to men (17). However, declining E2 levels during menopause correlate with an increase in cardiovascular disease (CVD) incidence (18).

In postmenopausal women, decreased endogenous E2 levels are associated with a higher risk of CVD, together with alterations in CVD risk factors and carotid artery remodeling (23).

The incidence of CVD shows a sex difference, resulting from the cardioprotective effects of endogenous estrogens. The incidence of ischemic heart disease, myocardial infarction, and sudden death is delayed by approximately 10 years in women (17). This protection diminishes during menopause, as declining E2 levels correlate with a higher incidence of CVD compared to premenopausal women, and conditions of premature estrogen deficiency, often seen after oophorectomy or due to other medical conditions, are linked to a markedly elevated risk of coronary artery disease than those without this deficiency (18).

### 5.2 Mechanistic basis linking menopause to cardiovascular disease

**Figure 3:**
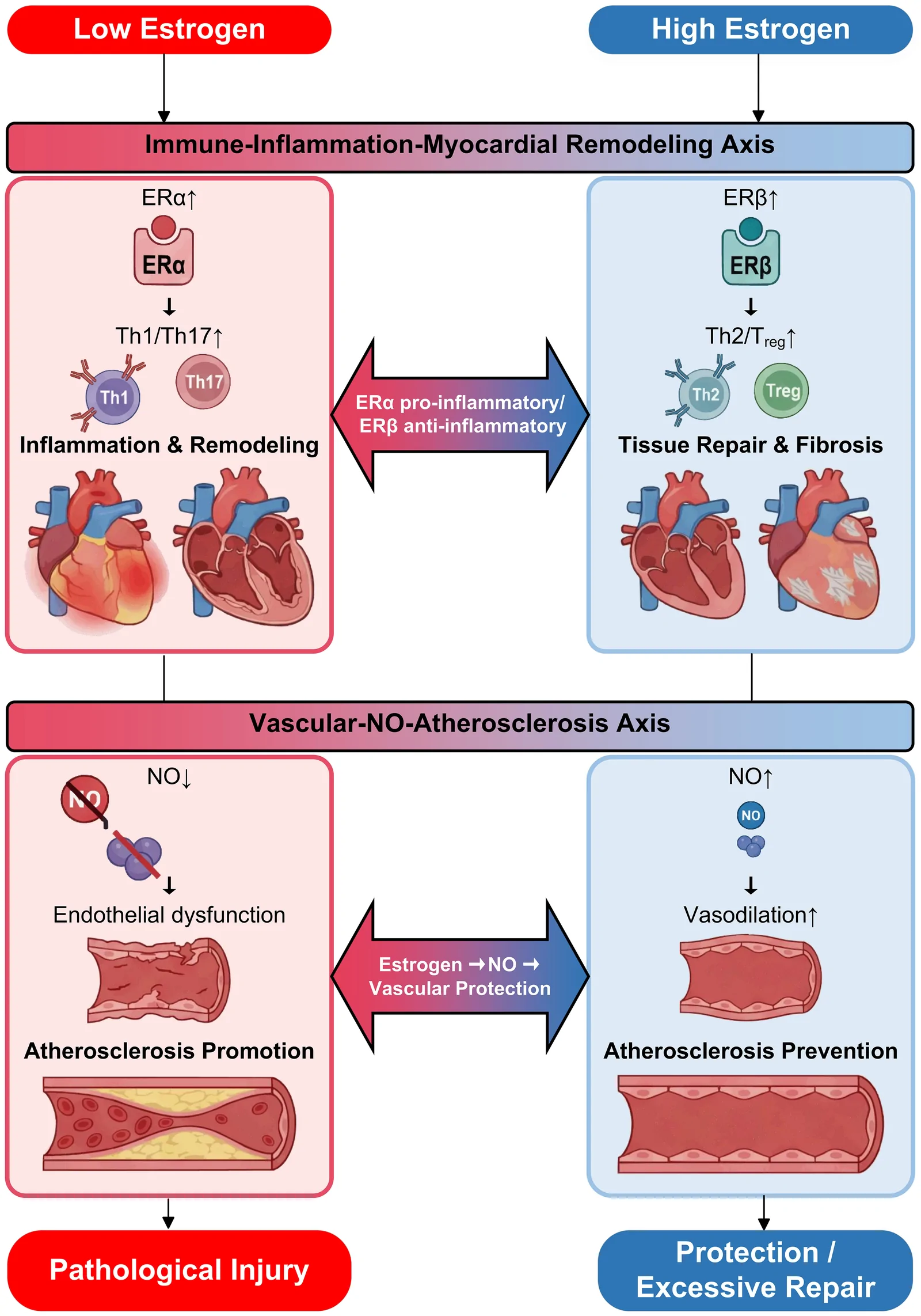
Bidirectional effects of estrogen on cardiovascular diseases in females. This schematic illustration explains how declining versus elevated estrogen (mainly E2) levels differentially regulate immune responses, myocardial remodeling, and vascular function. Under conditions of low estrogen (left), ERα-mediated signaling promotes polarization of CD4^+^ T cells toward proinflammatory Th1 and Th17 phenotypes, thereby enhancing cardiac inflammation and adverse myocardial remodeling. Concurrently, reduced NO bioavailability leads to endothelial dysfunction and promotes atherosclerosis, ultimately contributing to pathological cardiovascular injury. In contrast, high estrogen levels (right) favor ERβ-mediated signaling, driving CD4^+^ T-cell polarization toward anti-inflammatory Th2 and Treg phenotypes. This shift suppresses excessive inflammation and supports tissue repair. However, excessive activation may contribute to fibrosis. In the vasculature, estrogen enhances NO production, promoting vasodilation and protecting against atherosclerosis. The central concept highlights the dual and dynamic role of estrogen signaling: ERα is primarily associated with proinflammatory responses, whereas Erβ mediates anti-inflammatory and reparative processes. Together, these pathways define a balance between injury and protection/excessive repair during aging and menopause-related hormonal changes. E2, estradiol; ERα, estrogen receptor alpha; ERβ, estrogen receptor beta; Th1, T helper type 1 cell; Th2, T helper type 2 cell; Th17, T helper type 17 cell; Treg, regulatory T cell; NO, nitric oxide. Created by the authors based on Refs (17–27).

To explain the increased cardiovascular susceptibility after menopause, several interconnected mechanisms have been proposed. The protective and detrimental effects of estrogen signaling on cardiovascular homeostasis are summarized in **Figure 3**.

During cardiac inflammation, CD4+ T-cells are rapidly recruited to the heart, mediating the progression from acute injury to heart failure and driving adverse left-ventricular remodeling. This influx is associated with adverse left-ventricular remodeling and mediates the progression from acute cardiac injury to heart failure (19). While activated T cells facilitate the removal of cellular debris, CD4+ T cells dynamically fluctuate between proinflammatory and profibrotic phenotypes in chronic conditions like atherosclerosis (20). ER signaling dynamically modulates T-cell polarization to facilitate tissue repair; however, estrogen deficiency disrupts this balance, leading to pathological tissue remodeling (21).

In addition to immune regulation, estrogen deficiency directly impairs vascular endothelial function. Estrogen also binds to ERs on vascular smooth muscle cells, promoting increased nitric oxide (NO) release and vasomotor relaxation, thereby preventing atherosclerosis (21). Conversely, chronic estrogen deficiency following ovarian aging induces endothelial dysfunction and diminished NO bioavailability while also precipitating oxidative stress (22, 23). In the postmenopausal period, relative androgenicity or hyperandrogenemia leads to endothelial dysfunction and the development of atherosclerosis, partly by inhibiting the activation of endothelial NO synthase (eNOS) (24). While testosterone can increase reactive oxygen species (ROS) and oxidative stress, it also exhibits complex antioxidant effects. The antioxidative effect is maintained through conversion to E2, which helps regulate antioxidant enzymes and vasodilation (25).

Oxidative stress and chronic low-grade inflammation further accelerate cardiovascular injury during menopause. Aging endothelial cell (EC) and cardiomyocytes produce increasing levels of ROS, which impair vasorelaxation, increase vascular stiffness, and hinder EC turnover and NO release (26). Furthermore, menopause and aging are also associated with elevated levels of cytokines interleukin-1 (IL-1), IL-6, interleukin-18, TNF, and C-reactive protein (24, 27). Together, increased oxidative stress and chronic inflammatory cytokines accelerate endothelial injury, vascular remodeling, and atherosclerotic progression in postmenopausal women.

### 5.3 Clinical implications and hormone replacement therapy

Given the increased cardiovascular risk after menopause, HRT has been extensively investigated as a potential preventive strategy, although its cardiovascular benefits remain controversial. Multiple large epidemiological studies have demonstrated that HRT reduces the incidence of CVD in postmenopausal women (28). Clinical studies have also demonstrated that estrogen supplementation reduces vasoconstriction (29) and stress-related blood pressure (30) increases in perimenopausal and postmenopausal women, which are CVD risk factors. However, the 2015 Cochrane review concluded that HRT has little benefit in primary or secondary prevention and that it increases the risk of stroke and venous thromboembolic events (31). Larger randomized controlled trials have also failed to demonstrate any benefit in primary or secondary prevention (32).

The healthy endothelium hypothesis holds that HRT has beneficial effects on healthy endothelium but harmful effects in the presence of established atherosclerotic plaques (33). It appears that the timing of HRT initiation could play an important role in reducing CVD risk. Observational studies (34) and randomized controlled trials (35) have demonstrated that HRT could reduce all-cause mortality or coronary heart disease when started at the optimal time (especially under age 60 and at or near menopause). Therefore, current evidence suggests that individualized assessment, patient age, time since menopause, and baseline cardiovascular risk should be carefully considered when evaluating HRT for cardiovascular protection.

## 6 Menopause-related autoimmune diseases

### 6.1 Clinical significance of menopause-related hormonal changes in systemic lupus erythematosus and rheumatoid arthritis

Autoimmune diseases represent a leading cause of morbidity and mortality in women, driven largely by sexual dimorphism in immune responses (36). Menopause is accompanied by profound hormonal changes that alter immune homeostasis, thereby influencing both the incidence and clinical manifestations of autoimmune diseases, particularly systemic lupus erythematosus (SLE) and rheumatoid arthritis (RA).

There is evidence suggesting that early menopause, surgical menopause, and postmenopausal hormone use are all associated with an increased SLE risk, multiple studies have indicated that the incidence of SLE exhibits a bimodal pattern, with one peak occurring between the ages of 50 and 59 (43) (44).

In SLE patients, disease activity decreases during the postmenopausal phase, while cumulative damage increases, which may be related to age, disease duration, menopausal changes, the long-term effects of treatment, or a combination of the above (45). The LUMINA (Lupus in Minorities: Nature versus Nurture) investigators identified cyclophosphamide as the key factor underlying the increased age, longer disease duration, and higher damage scores at enrollment observed in menopausal SLE patients (46).

Late-onset RA (>60 years old) is associated with a more acute disease onset, more frequent involvement of larger proximal joints, more extensive systemic manifestations, higher ESR at onset, lower incidence of rheumatoid factor, and worse functional outcomes (47).

The weakened immunomodulatory effect of estrogen in menopause may be related to the adverse clinical outcomes of RA. Several studies have demonstrated that menopause is clinically relevant to the risk of RA development and worsening functional decline (37), as progression is often less favorable in postmenopausal women compared to premenopausal women (48).

Collectively, these clinical observations indicate that menopause-related hormonal alterations substantially influence autoimmune disease susceptibility and disease progression, providing a rationale for understanding the underlying biological mechanisms.

### 6.2 Mechanistic basis linking menopause to systemic lupus erythematosus and rheumatoid arthritis

To explain the altered susceptibility and clinical manifestations of autoimmune diseases after menopause, several interconnected mechanisms have been proposed.

**Figure 4:**
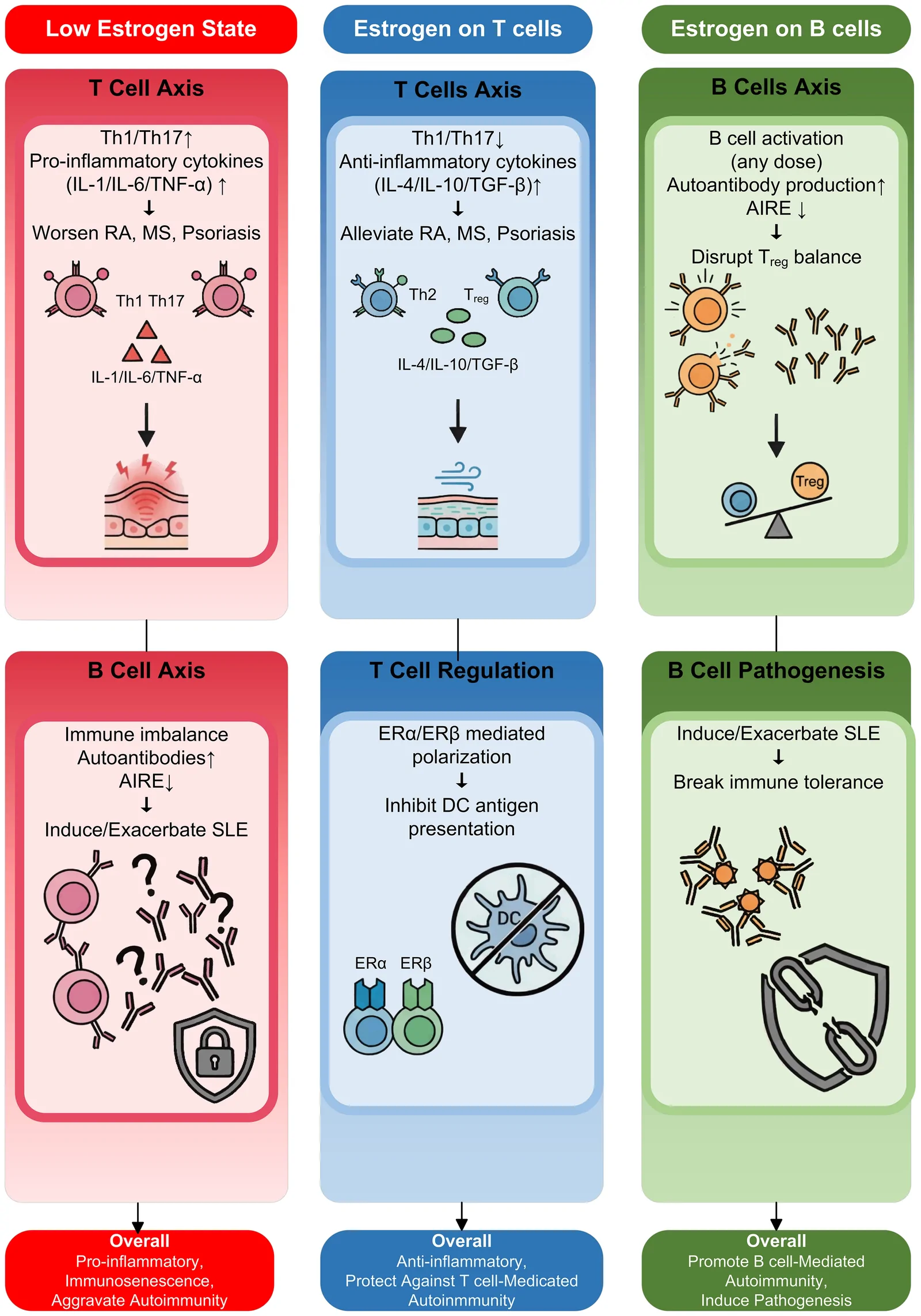
Immunomodulatory effect of estrogen on the T cell and B cell responses in autoimmune diseases of menopausal females. This schematic illustration explains the dual effects of estrogen on immune regulation during menopause. Under low estrogen conditions(left), CD4+ T cells shift toward proinflammatory Th1/Th17 phenotypes with increased cytokine production (IL-1, IL-6, TNF-α), while B-cell dysregulation and reduced AIRE expression promote autoantibody production, exacerbating autoimmune diseases. In contrast, normal or high estrogen signaling in T cells suppresses Th1/Th17 responses and enhances anti-inflammatory Th2 and Treg cells via ER-mediated pathways, alleviating inflammation. However, estrogen consistently stimulates B cells, enhancing activation and autoantibody production at any dose, disrupting Treg balance and promoting B cell–mediated autoimmunity, particularly SLE. Overall, menopausal estrogen decline promotes a proinflammatory and immunosenescent state, whereas estrogen exhibits cell-specific bidirectional immunomodulatory effects. AIRE, autoimmune regulator; DC, dendritic cell; ER, estrogen receptor; ERα, estrogen receptor alpha; ERβ, estrogen receptor beta; IL, interleukin; IL-4, interleukin-4; IL-6, interleukin-6; IL-10, interleukin-10; IL-11, interleukin-11; MS, multiple sclerosis; RA, rheumatoid arthritis; SLE, systemic lupus erythematosus; TGF-β, transforming growth factor beta; Th1, T helper type 1 cells; Th2, T helper type 2 cells; Th17, T helper type 17 cells; TNF-α, tumor necrosis factor alpha; Treg, regulatory T cells. Created by the authors based on Refs (27, 36–40).

Estrogen exerts dual, dose-dependent effects on the immune system: low levels (typical of menopause) promote T-cell-driven rheumatic diseases, whereas high levels suppress T helper 1/T helper 17 (Th1/Th17) responses. The differential effects of estrogen on T-cell and B-cell-mediated immune regulation are illustrated in **Figure 4**. Estrogens exert pro-inflammatory effects on B cells and anti-inflammatory effects on T cells under normal conditions. Low levels of endogenous estrogen, such as those associated with menopause, may increase the risk of developing T cell-driven autoimmune rheumatic diseases. However, at high concentrations, estrogen suppresses Th1 and Th17 cells via estrogen receptor-α and macrophages. Estrogen appears to promote the production of cytokines IL-4, IL-10, and transforming growth factor-beta (TGF-β) (37).

Despite its protective effects on T cells, estrogen continually stimulates B cells at varying concentrations. Any dose of estrogen may induce B-cell-dependent autoimmune diseases and B-cell-mediated autoimmunity, particularly in conditions like SLE, by enhancing B-cell survival and downregulating autoimmune regulator (AIRE) expression through promoter methylation. Downregulating AIRE expression may disrupt the delicate balance between autoreactive and regulatory T cells (38).

Multiple sclerosis (MS), rheumatoid arthritis (RA), psoriasis, and type 1 diabetes is considered Th1-mediated autoimmune diseases, whereas SLE is primarily Th2-mediated. During the perimenopause transition, the decline in estrogen levels is associated with immunological changes observed in multiple sclerosis, including increased secretion of pro-inflammatory cytokines, such as IL-1, IL-6, and TNF-α, and reduced production of anti-inflammatory cytokines (39). Estrogen typically exerts a protective effect by inhibiting the production of interleukin-12 and TNF-α, suppressing the antigen-presenting ability of dendritic cells, and stimulating the production of anti-inflammatory IL-10. In contrast, a decline in estrogen levels during menopause can exacerbate conditions like psoriasis and RA, whereas high E2 levels tend to shift the Th cell ratio from Th1 to Th2, relieving disease activity. Conversely, low estrogen levels can stimulate the release of cytokines that modulate Th2 responses, enhance natural killer cell activity, and stimulate B cells to produce antibodies (27), thereby promoting the occurrence of SLE (40).

Besides estrogen deficiency, androgen signaling may also modulate autoimmune susceptibility. Androgens have direct and indirect anti-inflammatory effects on the immune system (37, 41). Males exhibit a significantly lower risk of developing autoimmune rheumatic diseases than females (42). Furthermore, testosterone may exert a protective effect against SLE, and progesterone is generally immunosuppressive (37, 41).

Collectively, estrogen deficiency, immune-cell imbalance, B-cell activation, inflammatory cytokines, and altered androgen signaling interact to promote autoimmune dysregulation during menopause.

### 6.3 Clinical implications and hormone replacement therapy in systemic lupus erythematosus and rheumatoid arthritis

Traditionally, exogenous estrogen has been considered to potentially exacerbate the autoimmune process. Recent studies have shown that postmenopausal HRT is effective in most patients with rheumatic diseases (49).

Although research on the application of HRT in patients with SLE yields conflicting results, overall, no significant increase in disease activity is observed. In the Nurses Health Studies, the use of HRT for 2 years or longer was found to be associated with an increased risk of developing SLE; however, this finding may be biased by misattribution of SLE symptoms to menopause (44). In the large randomized controlled trial HRT-SELENA, HRT led to an increase in the incidence of mild to moderate SLE (50). Unlike the SELENA study, other studies, which did not subdivide flares by disease severity, demonstrated that the rate of flares with HRT is not increased compared with that in the placebo group (51).

The effects of HRT on RA are also contradictory. While some studies, such as the WHI study, noted no significant reduction in the risk of RA in the use of estrogen or estrogen plus progestin (52), multiple studies have shown that HRT has a significant positive effect on bone density and osteoporosis in RA patients (53). HRT during menopause may have a protective effect in RA patients dominated by T cells. Previous studies have shown that HRT can reduce both RA risk and disease activity (54). However, other studies have not found any significant correlation (55). One study indicated that HRT does not increase the incidence of coronary artery disease in RA patients (56); however, data on this effect remain limited.

Overall, current evidence suggests that HRT may be considered on an individualized basis after carefully balancing disease activity, thrombotic risk, cardiovascular risk, and menopausal symptoms.

## 7 Menopausal transition and the disruption of neuroendocrine homeostasis

### 7.1 Clinical significance of menopause-related hormonal changes in neurodegenerative diseases

The brain is a primary target for sex hormones, which regulate glucose metabolism, mitochondrial bioenergetics, and neuronal survival. Postmenopausal women experience a relatively rapid decline in ovarian hormone levels, which becomes a significant age-related risk factor for neurodegenerative diseases, such as AD.

Cerebral immune activation and metabolic decline are highly coupled and occur concurrently during the menopausal transition. Neuroimaging and experimental studies show that the perimenopausal stage is characterized by reductions in cerebral glucose metabolism, mitochondrial respiration, and white-matter integrity, accompanied by increased β-amyloid deposition and alterations in neural function (63). As brain glucose utilization declines, there is a compensatory shift toward alternative energy sources, including fatty acid metabolism and ketone bodies (66). This transition leads to increased oxidative stress, lipid peroxidation, and mitochondrial dysfunction, ultimately generating damage-associated molecular patterns (DAMPs) such as extracellular ATP, mitochondrial DNA, and reactive oxygen species that further amplify chronic inflammatory signaling (67).

AD and PD represent the two most prevalent neurodegenerative disorders, yet they exhibit starkly different sex-specific clinical profiles. AD is the most common cause of dementia, accounting for 60%–70% of all cases. Compared with men, middle-aged women face a 2–3 times higher risk of developing AD and tend to progress more severely (70). In contrast to AD, PD has a higher incidence rate in males than in females, and the age of PD onset is generally also earlier in men (71). Male patients experience more severe disease progression, confirmed in animal models of PD (72). Furthermore, PD-associated gene expression in the substantia nigra and striatum and dopamine metabolism also exhibit sex differences in PD brains (73).

Collectively, epidemiological, clinical, and neuroimaging evidence suggests that menopause-related hormonal changes substantially increase the risk and progression of neurodegenerative diseases.

### 7.2 Mechanistic basis linking menopause to neurodegenerative diseases

To explain these clinical observations, several interconnected mechanisms have been proposed.

**Figure 5:**
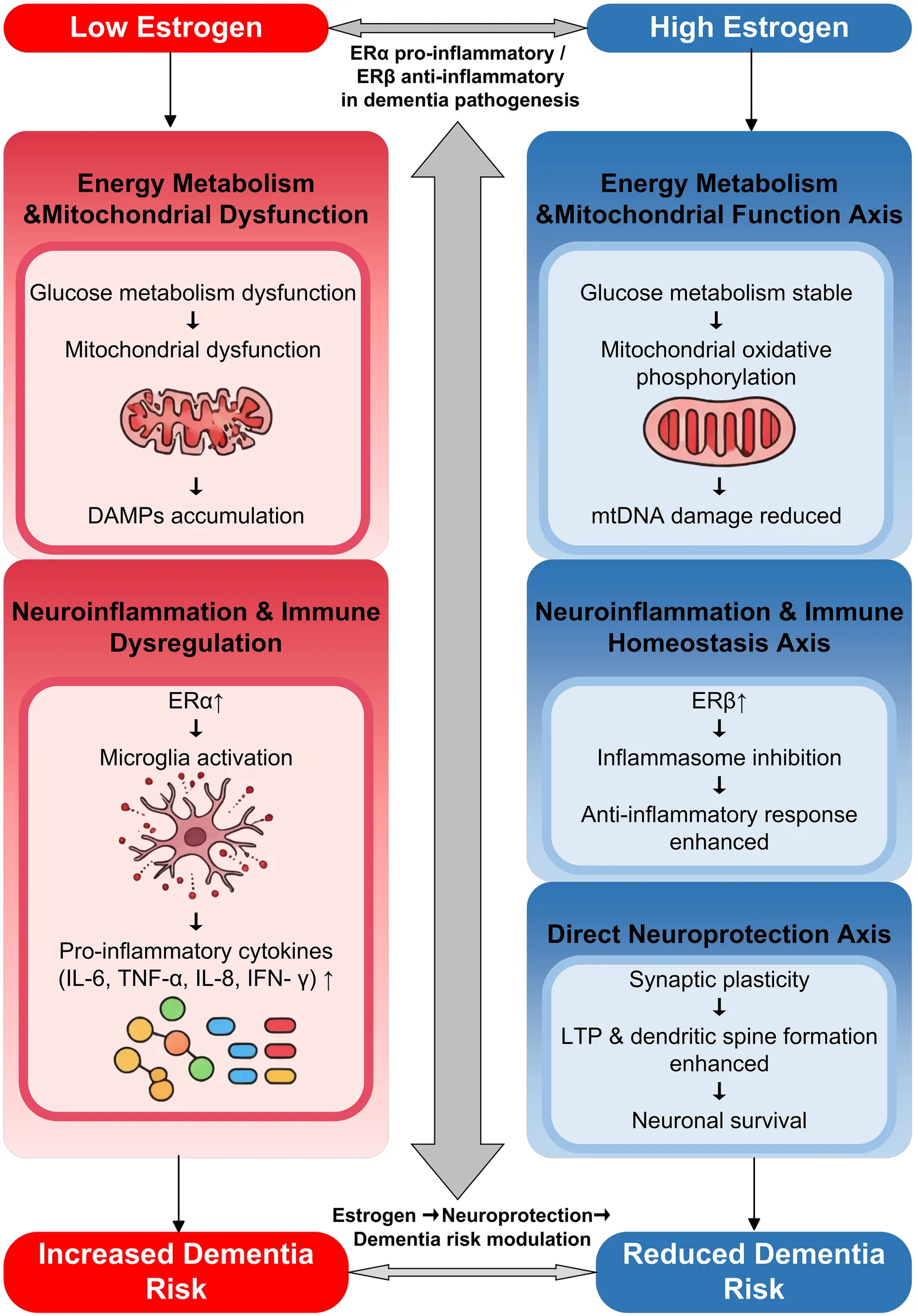
Bidirectional effects of estrogen on neuroinflammation and dementia risk in females. This schematic illustration explains the bidirectional effects of estrogen on brain metabolism, neuroinflammation, and neuroprotection during menopause. Under low estrogen conditions (left), impaired glucose metabolism and mitochondrial dysfunction lead to the accumulation of DAMPs. Also, low estrogen level promotes ERα signaling, triggering microglial activation (CD14, TLR4, C3) and increased production of proinflammatory cytokines (IL-6, TNF-α, IL-8, IFN-γ). This proinflammatory state contributes to chronic neuroinflammation, synaptic dysfunction, and increased dementia risk. In contrast, high estrogen level (right) maintains mitochondrial oxidative phosphorylation and energy homeostasis, reducing mtDNA damage and supporting ATP production. Improved ERβ signaling suppresses inflammasome activation and enhances Treg-mediated anti-inflammatory responses. Additionally, estrogen promotes synaptic plasticity, LTP, dendritic spine formation, and neuronal survival, thereby exerting direct neuroprotective effects and reducing dementia risk. Overall, estrogen-dependent signaling, particularly the balance between ERα-mediated proinflammatory and ERβ-mediated anti-inflammatory pathways, determines the transition between neurodegeneration and neuroprotection during menopause. ATP, adenosine triphosphate; DAMPs, damage-associated molecular patterns; ERα, estrogen receptor alpha; ERβ, estrogen receptor beta; IFN-γ, interferon gamma; IL-6, interleukin-6; IL-8, interleukin-8; TLR4, Toll-like receptor 4; Treg, regulatory T cell; LTP, long-term potentiation; mtDNA, mitochondrial DNA; TNF-α, tumor necrosis factor alpha. Created by the authors based on Refs (27, 57–59, 63–67).

In the brain, estrogen functions as a critical regulator of glucose metabolism, mitochondrial bioenergetics, and neuronal survival. The proposed mechanisms linking estrogen deficiency with metabolic dysfunction, neuroinflammation, and neurodegeneration are summarized in **Figure 5**. Estrogen dysregulation triggers glucose metabolism and mitochondrial oxidative phosphorylation (57). These processes may contribute to neurodegenerative vulnerability. In addition to metabolic regulation, estrogen exerts immunomodulatory effects in both the central and peripheral nervous systems (27).

ERα and ERβ are abundantly expressed in astrocytes, microglia, and neurons. In murine models of MS, the selective activation of ERβ modulates microglia-mediated neuroinflammation and protects oligodendrocytes from demyelination, whereas selective activation of ERα also exhibits neuroprotective effects mediated by astrocytes (58).

Sex hormones, including E2, androgens, and progestogens, exhibit neuroprotective properties. The neuroprotective action of E2 has been extensively studied across multiple experimental models of neurodegenerative diseases. This effect is particularly pronounced in ischemic brain injury. Epidemiological observations also support the neuroprotective role of sex hormones. E2 exerts its effects through both genomic and rapid non-genomic signaling pathways. It influences neurogenesis, immune responses within the central nervous system, and resistance to excitotoxic injury (59).

Androgens also contribute to neuroprotection. Testosterone and its metabolites can protect hippocampal neurons and astrocytes from metabolic stress and oxidative damage. In addition, testosterone prevents dendritic atrophy in motoneurons following peripheral nerve injury and may support cognitive function through androgen receptor signaling in hippocampal neurons (60). In addition to direct neuronal effects, progesterone modulates neuroinflammation by reducing inflammatory mediators and limiting microglial activation (61). In the EAE mouse model, progesterone treatment has been associated with reduced clinical symptoms, decreased demyelination, and improved neuronal function, indicating that the reduction of neuroinflammation partially mediates the beneficial effects of progesterone in the EAE model (62). Progesterone may also influence glial cell function and oligodendrocyte maturation, processes that are important for myelin maintenance and repair (61). Together, these findings support the view that progesterone acts as a multifunctional neurosteroid capable of promoting neuroprotection and modulating inflammatory responses within the central nervous system. During midlife, both chronological aging and endocrine aging activate chronic inflammatory processes, ultimately limiting microglial clearance capacity and leading to immune senescence. The endocrine transition of perimenopause to post-menopause is accompanied by the loss of ovarian steroid hormones and the emergence of chronic low-grade inflammation, which is increasingly recognized as a systemic feature of reproductive aging (63). These inflammatory processes influence both peripheral and central immune regulation and may accelerate ovarian functional decline while altering neuroimmune signaling pathways.

The abrupt decline in ovarian steroid hormones during menopause significantly impairs the immunomodulatory capacity of the central nervous system (64). An analysis of microarray data from National Center for Biotechnology Information (NCBI) of inflammatory gene expression profiles in the postcentral and superior frontal gyrus showed that postmenopausal women exhibited upregulation of the microglial markers CD14, CD18, and CD45, as well as Toll-like receptor 4 (TLR4) and major histocompatibility complex class II (MHC-II) markers CD74 and complement component 3 (C3), compared with premenopausal women (65). Evidence from human transcriptomic data and ovariectomized animal models consistently demonstrates that hormone deprivation triggers a functional shift in microglia from a homeostatic state toward a reactive phenotype. This transition is characterized by the robust upregulation of markers associated with phagocytosis, the complement system, and antigen presentation (e.g., CD45, TLR4, MHC-II, and C3), signaling a profound shift toward a pro-inflammatory microenvironment within the brain (65).

In addition to serving as a target for sex hormones, the brain has been identified as a steroidogenic organ with compensatory mechanisms that counteract peripheral hormone deficiencies (59). Like peripheral sex hormones, neurosteroids have also been demonstrated to exert neuroprotective actions. Furthermore, endogenous neurosteroids may enhance the protective effects of exogenous steroids (68). Indeed, the estrogen levels in the brain have been shown to determine the protective efficacy of estrogen therapy against AD pathology in mice (68). As neurosteroids can be converted from peripheral steroid hormones in the blood into derivatives with potent neuromodulatory actions, fluctuations in plasma steroid levels are expected to influence the rate of brain steroid synthesis (69).

Collectively, alterations in energy metabolism, neuroimmune regulation, microglial activation, and neurosteroid synthesis jointly contribute to neurodegeneration after menopause.

### 7.3 Clinical implications and hormone replacement therapy in neurodegenerative diseases

Given the increased risk of neurodegenerative diseases after menopause, HRT has been extensively investigated as a potential strategy to preserve cognitive function and delay disease progression.

Estrogens, particularly E2, exhibit neuroprotective effects in both AD and PD models (74). However, the precise contribution of ER subtypes to AD neuroprotection remains unclear. Observational studies in postmenopausal women have indicated that those who receive HRT exhibit a reduced risk of PD, although findings remain inconsistent (75). Furthermore, compared with late-onset PD patients, early-onset patients more commonly exhibit polymorphisms in the human ERβ gene in both sexes (76). In contrast, ERα polymorphisms appear unrelated to PD (77). Moreover, testosterone cannot efficiently aromatize to exert neuroprotective effects in PD mouse models, or it exerts effects opposite to those of E2 in the brain (78).

HRT promotes neuronal survival, and it has been shown to improve cognitive function and episodic memory in perimenopausal and postmenopausal women (79). The findings of numerous preclinical and epidemiological studies, and some clinical trials, support the beneficial effects of HRT on memory and cognition and reduced risk for AD (80). Consistent with a “window of opportunity” hypothesis, women transitioning through menopause derive more benefit from HRT than those who have already transitioned (81). This effect of E2 in HRT can be explained by the healthy cell bias, which emphasizes the role of neuronal viability and health at baseline on E2 to exert its therapeutic effects (79), and critical window hypothesis, which focus on the perimenopausal transition as a key phase for HRT use when cells remain healthy (82).

Like E2, selective estrogen receptor modulators (SERMs), such as tamoxifen, exert neuroprotective effects by reducing neuroinflammation (83).

Notably, combined hormone therapy involving E2 and progestin does not appear to produce the same neuroprotective effects as E2 alone (84). Progestin may antagonize the protective effects of E2.

Overall, current evidence supports that HRT can alleviate depressive symptoms and cognitive decline and may reduce the risk of dementia; however, there is also evidence that HRT may increase dementia risk (85).

## 8 Endocrine reprogramming and the evolution of the pro-tumorigenic microenvironment

### 8.1 Clinical significance of menopause-related hormonal changes in cancer

Menopause, characterized by a decline in E2 and progesterone alongside a relative increase in circulating androgens and gonadotropins—serves as a critical biological transition that reshapes the oncogenic landscape. Beyond the direct proliferative effects on hormone-dependent tissues, current research emphasizes that menopausal hormonal shifts act as a systemic “master switch” that alters immune surveillance, metabolic homeostasis, and the inflammatory state of the tumor microenvironment (TME).

The clinical relevance of mechanisms is particularly evident in breast cancer. More than 70% of primary breast cancers are estrogen receptor–positive (ER+), and these tumors exhibit estrogen-dependent growth. During neoplastic transformation, ER+ epithelial cells shift from paracrine to autocrine estrogen signaling, promoting cellular proliferation and tumor development. Accordingly, endocrine therapies targeting estrogen signaling have significantly reduced recurrence risk in patients with ER+ breast cancer, highlighting the central role of estrogen signaling in breast cancer progression and providing a strong rationale for therapeutic intervention (90).

Estrogen has been found to have protective effects in some experimental models of colorectal cancer (CRC), although its role remains complex and context dependent. Experimental and molecular studies indicate that estrogen signaling can inhibit CRC cell proliferation through mechanisms such as modulation of DNA repair pathways, regulation of microRNAs, and cell-cycle control; however, E2 may also promote oncogenic signaling under certain conditions depending on receptor subtype activation and tumor stage (91).

Epidemiological evidence examining endogenous sex hormones and CRC risk in women has produced inconsistent findings. Large prospective analyses of postmenopausal women have generally reported no clear association between circulating concentrations of E2 or estrone and CRC risk, suggesting that endogenous estrogen levels alone may not be a strong determinant of disease development (92). Nonetheless, hormonal signaling in colonic tissue may still influence tumor progression through differential activation of estrogen receptor subtypes during tumor evolution.

Collectively, these epidemiological and clinical observations indicate that menopause-related hormonal alterations influence both tumor susceptibility and tumor progression, particularly in hormone-responsive cancers.

### 8.2 Mechanistic basis linking menopause to tumor development

**Figure 6:**
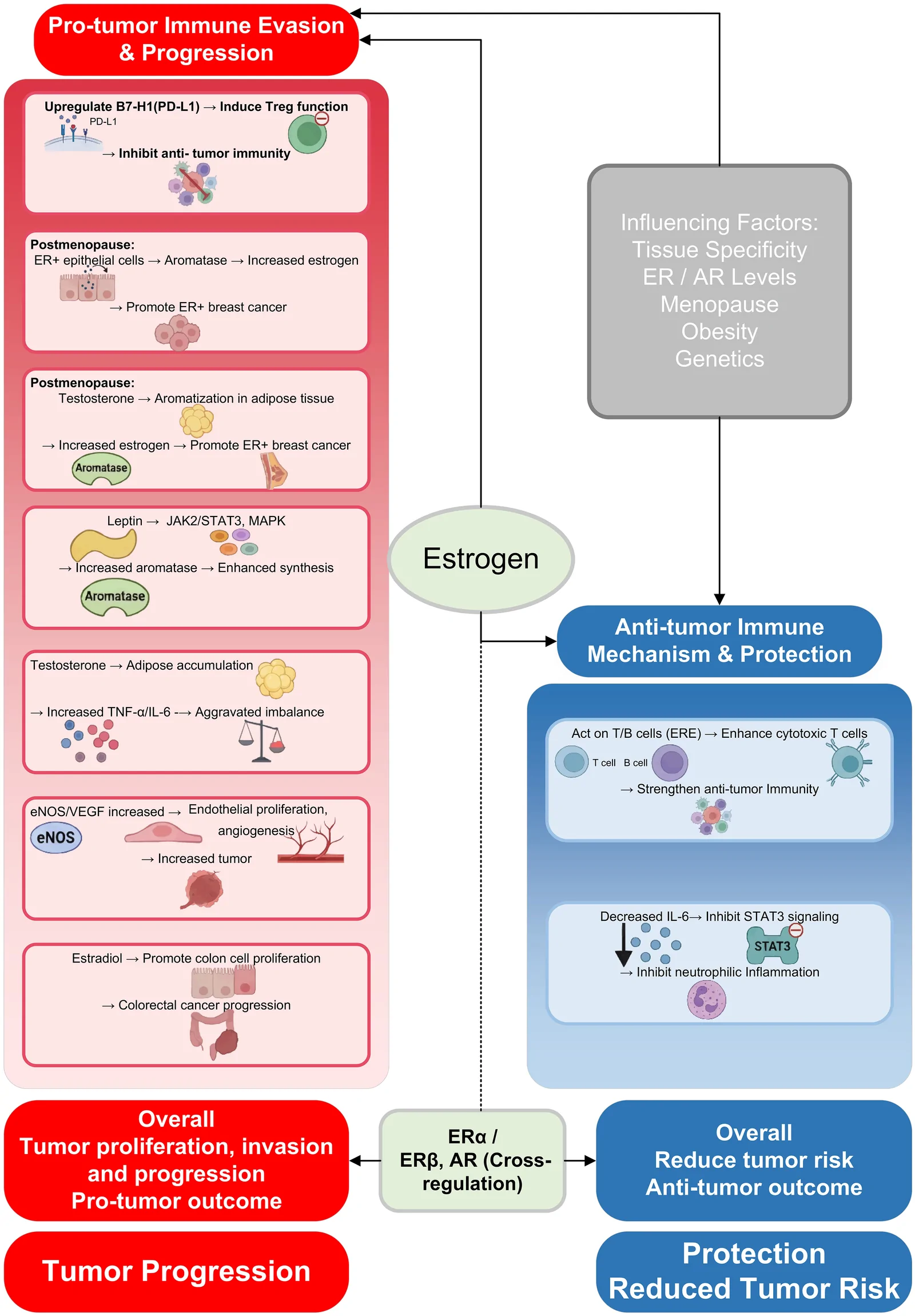
Bidirectional effects of estrogen on tumor development and progression in menopausal females. This schematic illustration explains the context-dependent dual effects of estrogen on tumor immunity and cancer progression. Through ERα/ERβ and AR cross-regulation, estrogen is modulated by tissue specificity, receptor expression, menopause, obesity, and genetic factors. On the pro-tumor side(left), estrogen promotes immune evasion by upregulating B7-H1 (PD-L1) and inducing Treg cell activity. Postmenopausal hormonal imbalance increases aromatase activity and adipose-derived estrogen production, promoting ER+ tumor growth. Leptin signaling (JAK2/STAT3, MAPK) and inflammatory cytokines (TNF-α, IL-6) further exacerbate tumor-promoting pathways. Estrogen also enhances endothelial proliferation and angiogenesis via eNOS/VEGF signaling and may stimulate colorectal cancer cell proliferation. Conversely, on the anti-tumor side (right), estrogen enhances cytotoxic T cell and B cell responses, strengthens antitumor immunity, and suppresses neutrophilic inflammation via inhibition of IL-6–STAT3 signaling, contributing to reduced tumor risk. Overall, these mechanisms drive either tumor protection or tumor proliferation and progression depending on the biological context. AR, androgen receptor; eNOS, endothelial nitric oxide synthase; ER, estrogen receptor; ERα, estrogen receptor alpha; ERβ, estrogen receptor beta; ER+, estrogen receptor positive; ERE, estrogen response element; IL-6, interleukin-6; JAK2, Janus kinase 2; MAPK, mitogen-activated protein kinase; PD-L1, programmed death-ligand 1; STAT3, signal transducer and activator of transcription 3; TNF-α, tumor necrosis factor alpha; Treg, regulatory T cells; VEGF, vascular endothelial growth factor. Created by the authors based on Refs (86–118).

To explain how menopausal hormonal alterations reshape tumor biology, several interconnected mechanisms have been proposed. The dual roles of estrogen signaling in tumor immune regulation and tumor progression are summarized in **Figure 6**.

#### 8.2.1 Immune dysregulation and tumor immune escape

Immune dysregulation is one of the earliest events contributing to the establishment of a pro-tumorigenic microenvironment after menopause.

The postmenopausal decline in estrogen disrupts the robust immune surveillance typically observed in females. In the innate immune system, estrogen deficiency impairs the antigen-presenting efficiency of dendritic cells and macrophages (86). Affected by both chromosomal and sex hormone factors, innate and adaptive immune responses exhibit gender differences, with females generally demonstrating stronger overall immune responses (86).

Sexual dimorphism in cancer susceptibility is driven by the potent immunomodulatory effects of estrogen on both innate and adaptive surveillance. In the innate immune system, cancer cells can evade immune destruction by modulating antigen presentation, whereas antigen-presenting cells (APCs) present peptide fragments more efficiently in females than in males. However, estrogen signaling can paradoxically facilitate immune evasion by upregulating checkpoint molecules such as PD-L1 on APCs and tumor cells, which reinforces regulatory T-cell activity while blunting anti-tumor immunity (87). Furthermore, estrogen has been shown to suppress IL-6–mediated inflammatory amplification and neutrophil-driven inflammation in oncogenic contexts (88). Within the adaptive immune system, estrogen exerts direct genomic control; many T-cell genes contain estrogen response elements (EREs) in their promoters, including antiviral genes such as IFNG, OAS1, and pro-inflammatory genes such as IL12RB2 and IL16, which are associated with stronger cytotoxic T-cell responses and enhanced anti-tumor immune surveillance in females (86, 89).

#### 8.2.2 Estrogen receptor signaling and tumor cell reprogramming

In addition to its systemic immunomodulatory effects, the estrogen signaling pathway also contributes to the initiation and progression of female reproductive system tumors by shaping the tumor immune microenvironment, with particularly important roles in breast cancer, ovarian cancer, and endometrial cancer. Estrogen primarily exerts its biological effects through genomic and non-genomic mechanisms mediated by estrogen receptors ERα and ERβ (133). ERα predominantly promotes cell proliferation, whereas ERβ exerts antiproliferative effects and contributes to the maintenance of tissue homeostasis (133, 134). An imbalance in the ERα/ERβ expression ratio in breast, ovarian, and fallopian tube tissues, particularly the reduced expression of ERβ, has been closely associated with tumorigenesis (135, 136).

Activated ERα directly regulates the transcription of multiple proto-oncogenes and cell cycle-related genes, including c-fos, c-myc, HER2/neu, cyclins, and various growth factors, thereby driving sustained tumor cell proliferation and inhibiting apoptosis (137). Meanwhile, estrogen promotes local angiogenesis and facilitates the establishment of an immunosuppressive tumor microenvironment, thereby creating favorable conditions for immune evasion and tumor progression (109, 110). In addition, the reactive oxygen free radicals produced during estrogen metabolism may exert mutagenic effects and further contribute to genomic instability, thereby enhancing tumor initiation and development (133, 135).

In endometrial cancer, the carcinogenic effects of estrogen are primarily manifested through sustained activation of a pro-inflammatory tumor immune microenvironment. Estrogen can induce the production of inflammatory mediators, including IL-6, IL-1, TNF-α, and matrix metalloproteinases, while activating the NF-κB signaling pathway (11, 138, 139). Consequently, the expression of angiogenic and pro-inflammatory genes, such as VEGF and IL-8, is enhanced, leading to increased leukocyte infiltration, extracellular matrix degradation, tumor angiogenesis, and ultimately accelerated tumor invasion and progression (110, 139). Sustained activation of NF-κB signaling establishes a positive feedback loop with estrogen-driven inflammatory signaling, thereby maintaining a pro-inflammatory microenvironment that further facilitates the initiation and progression of endometrial cancer (140).

#### 8.2.3 Metabolic-androgenic reprogramming

Postmenopausal women often undergo a metabolic shift toward visceral adiposity, which functions as a secondary endocrine organ, creating a “vicious cycle” of oncogenesis. Visceral adipose tissue produces pro-inflammatory cytokines such as TNF-α and IL-6, which can activate signaling pathways that promote insulin resistance and tumor-promoting inflammation. These metabolic disturbances may interact with altered sex hormone signaling, contributing to colorectal carcinogenesis (93).

Testosterone promotes adipose tissue accumulation, coupled with ovarian estrogen imbalance, leading to overweight, which is commonly observed in postmenopausal women. Increased adipose tissue induces insulin resistance, particularly central obesity. Accumulating evidence indicates that visceral adiposity induces the production of proinflammatory cytokines, such as TNF-α and IL-6, which promote other inflammatory cytokines and trigger cellular signaling pathways that lead to insulin resistance (94, 95).

In the context of obesity, this low-grade chronic inflammation leads to the dysregulation of immune cells, cytokines, and adipokines, thereby promoting a pro-carcinogenic environment (96).

Moreover, a recent large meta-analysis confirmed that postmenopausal women who are overweight or obese face a 1.5-fold increased risk of developing ER+/Progesterone receptor–positive (PR+) breast cancer, respectively, compared with normal-weight women (97). In addition, an abnormal increase in adiposity is associated with an increased risk of distant recurrence (98). Moreover, clinical data indicate that obesity is associated with poorer outcomes and a higher likelihood of recurrence among patients with hormone receptor–positive breast cancer receiving endocrine therapy (99). Clinical studies have indicated that most patients eventually develop resistance to aromatase inhibitors (AIs) and experience disease progression. The efficacy of adjuvant endocrine treatment depends on the total volume of adipose tissue and the consequent overexpression of aromatase (100). Conflicting evidence has emerged regarding the efficacy of AIs in women who are overweight or obese, suggesting that obesity adversely affects AI treatment responses and may contribute to drug resistance (101). The molecular mechanisms proposed by Ambrosio et al. elucidated how adipose tissue may promote breast cancer progression and indicated that interleukin-8 and its receptor may be potential therapeutic targets for overcoming tamoxifen resistance (102).

In postmenopausal women, elevated circulating testosterone levels enhance estrogen production through testosterone aromatization in adipose tissue, which provides a suitable microenvironment for the initiation and progression of breast cancer in ER+ epithelial cells (96). This process correlates positively with an increased risk of breast cancer. Additionally, in estrogen-depleted environments or during endocrine therapy, tumor cells with high AR expression may maintain proliferative signaling through alternative pathways, including interactions with the insulin/insulin-like growth factor (IGF) axis (103).

Experimental research suggests that testosterone can influence tumor growth through androgen receptor signaling pathways in colon cancer cells, which can be inhibited by anti-androgens (104). However, clinical studies remain inconclusive regarding whether circulating testosterone directly promotes CRC development (92).

Leptin is primarily produced and released by mature adipocytes in an insulin-dependent manner, and its circulating levels are directly correlated with body fat mass (105). Increasing evidence indicates that leptin plays a pivotal role in the “obesity-insulin-testosterone connection.” Over 80% of primary tumors produce leptin, and experimental studies have confirmed that leptin produced by cancer cells can promote the growth of neighboring cells in a paracrine manner by activating Janus kinase 2 (JAK2)/STAT3 and mitogen-activated protein kinase (MAPK) signaling pathways, thereby inducing aromatase gene expression. This effect promotes the production of bioactive estrogens by aromatizing androgens (106).

Breast cancer is potentially associated with iodine metabolism. Both human breast tissue and the thyroid gland are capable of iodide uptake, partly mediated by the sodium/iodide symporter, which is expressed in mammary tumors as well as thyroid tissue (107). Alterations in iodide handling have been observed in both malignant and normal breast tissues, suggesting a shared endocrine regulatory axis (107). In addition, breast cancer patients exhibit a higher prevalence of various thyroid disorders, and a potential association between low levels of free thyroxine (FT4) and breast cancer detection rates in perimenopausal and postmenopausal women (108).

#### 8.2.4 Angiogenesis and tumor microenvironment remodeling

While systemic vascular health declines post-menopause, the tumor-specific “angiogenic switch” can be triggered by fluctuations in E2 and progesterone. E2 stimulates EC proliferation and migration through ER-mediated phosphoinositide 3-kinase (PI3K)/protein kinase B (Akt) activation. In hypoxic TMEs, the interplay between residual estrogen and Vascular Endothelial Growth Factor (VEGF) promotes the formation of disorganized, leaky vessels that facilitate hematogenous metastasis (109, 110).

Sex hormones are important regulators of vascular biology and angiogenesis. Both ECs and endothelial progenitor cells express sex hormone receptors, including ERs, PRs and ARs. Estrogen, particularly E2, has been shown to stimulate EC proliferation and migration and to activate eNOS through ER-mediated signaling pathways (111). Experimental studies also demonstrate that E2 can enhance EC proliferation and migration under stress conditions such as hypoxia, suggesting a potential role for estrogen signaling in regulating angiogenic responses (112).

E2 also mediates EPC mobilization and influences diverse cells in the perivascular environment, including tumor cells (113). In the tumor setting, estrogen signaling can further enhance a pro-angiogenic microenvironment through tumor cell and stromal cell interactions and ER expression correlates positively with vascularization activity, tumor volume, and invasiveness in hormone-responsive malignancies (113, 114). This finding aligns with estrogen-mediated pro-angiogenic effects. By contrast, androgens do not exert proliferative, migratory, or angiogenic effects on female ECs (115).

Aging is another important factor influencing cancer development. The incidence of cancer increases exponentially with age, attributed to the accumulation of genetic mutations and changes in the immune system (116). Inflammaging is defined as a chronic low-grade inflammatory state induced by aging, and it is believed to arise from the various inflammatory cytokines secreted by proliferating senescent cells in various organs. Multiple stimuli can induce the senescence-associated secretory phenotype (SASP), a major driver of inflammaging in aging and age-related diseases, such as cancer (117). During aging, the physiological decline in sex hormones may play a role in cellular senescence. Recent laboratory evidence indicates that genetic or pharmacological inhibition of androgen signaling can induce a SASP with tumor-enhancing properties in dermal fibroblasts from both sexes and in fibroblasts associated with cutaneous neoplastic lesions. However, androgen stimulation produces the opposite beneficial effect (118).

### 8.3 Clinical implications and hormone replacement therapy in hormone-responsive cancers

Given the profound influence of menopausal hormonal changes on tumor biology, the safety and therapeutic implications of HRT remain an important clinical concern.

HRT partially reverses the effects of aging on immunity by increasing B-cell and T-cell counts and reducing the levels of proinflammatory cytokines (TNFα and IL-6) in postmenopausal women (119).

Furthermore, in women, HRT using a combination of estrogen and a progestogen may increase cancer risk in hormone-responsive tissues like the breast to varying degrees, depending on the type of progestogen used. However, when estrogen is combined with a natural progesterone or used alone, the breast cancer risk is decreased rather than increased (120). Evidence regarding ovarian cancer risk remains heterogeneous. A meta-analysis indicates that HRT use may influence ovarian cancer incidence depending on duration and formulation (121). Epidemiological studies have indicated that HRT is associated with a significant reduction in ovarian cancer risk, even in carriers of the breast cancer 1 (BRCA1) and breast cancer 2 (BRCA2) gene mutations (122).

In contrast, evidence from a systematic review indicates that the effect of menopausal hormone therapy on endometrial cancer is highly formulation-dependent. Estrogen-only therapy is associated with an increased risk of endometrial cancer in women with an intact uterus, whereas continuous combined estrogen–progestogen therapy is associated with a reduced risk of endometrial cancer. Sequential combined regimens may be associated with an increased risk, while the effect of micronized progesterone-containing regimens varies depending on dose, regimen, and duration of use (141).

Observational studies of the association between HRT and CRC risk in postmenopausal women have indicated that both former use of combined estrogen–progestogen therapy and current use of estrogen alone exhibit a negative correlation with disease incidence (123). A protective effect of combined hormone therapy was confirmed in the WHI estrogen plus progestin therapy clinical trial (124).

## 9 Menopause-related osteoporosis

### 9.1 Clinical significance of menopause-related hormonal changes in osteoporosis

Post-menopausal osteoporosis is the most prevalent form of osteoporosis, which results from decreased estrogen levels in menopause. The deficiency of estrogen can trigger a rapid phase of bone loss mainly in the trabecular bone, with an annual loss rate of approximately 3% to 5% in the first 5 to 10 years (125).

Following menopause, the sharp decline in estrogen accelerates bone remodeling and causes it to shift towards a state dominated by osteoclast activity (142).

Other hormones also participate in bone metabolism. FSH can activate the pathways responsible for bone remodeling, while PTH can regulate calcium metabolism (143). However, the high levels of these hormones may indicate a decrease in ovarian estrogen production and may contribute to bone remodeling dysfunction (143). In addition, the lack of progesterone secretion directly leads to bone loss (144). Postmenopausal progesterone therapy is ineffective for bone health, but progesterone and estradiol have an additive effect on bone health (144).

Collectively, menopause-related hormonal alterations profoundly disturb bone homeostasis and represent the principal endocrine driver of postmenopausal osteoporosis.

### 9.2 Mechanistic basis linking menopause to osteoporosis

To explain the accelerated bone loss after menopause, several interconnected mechanisms have been proposed.

**Figure 7:**
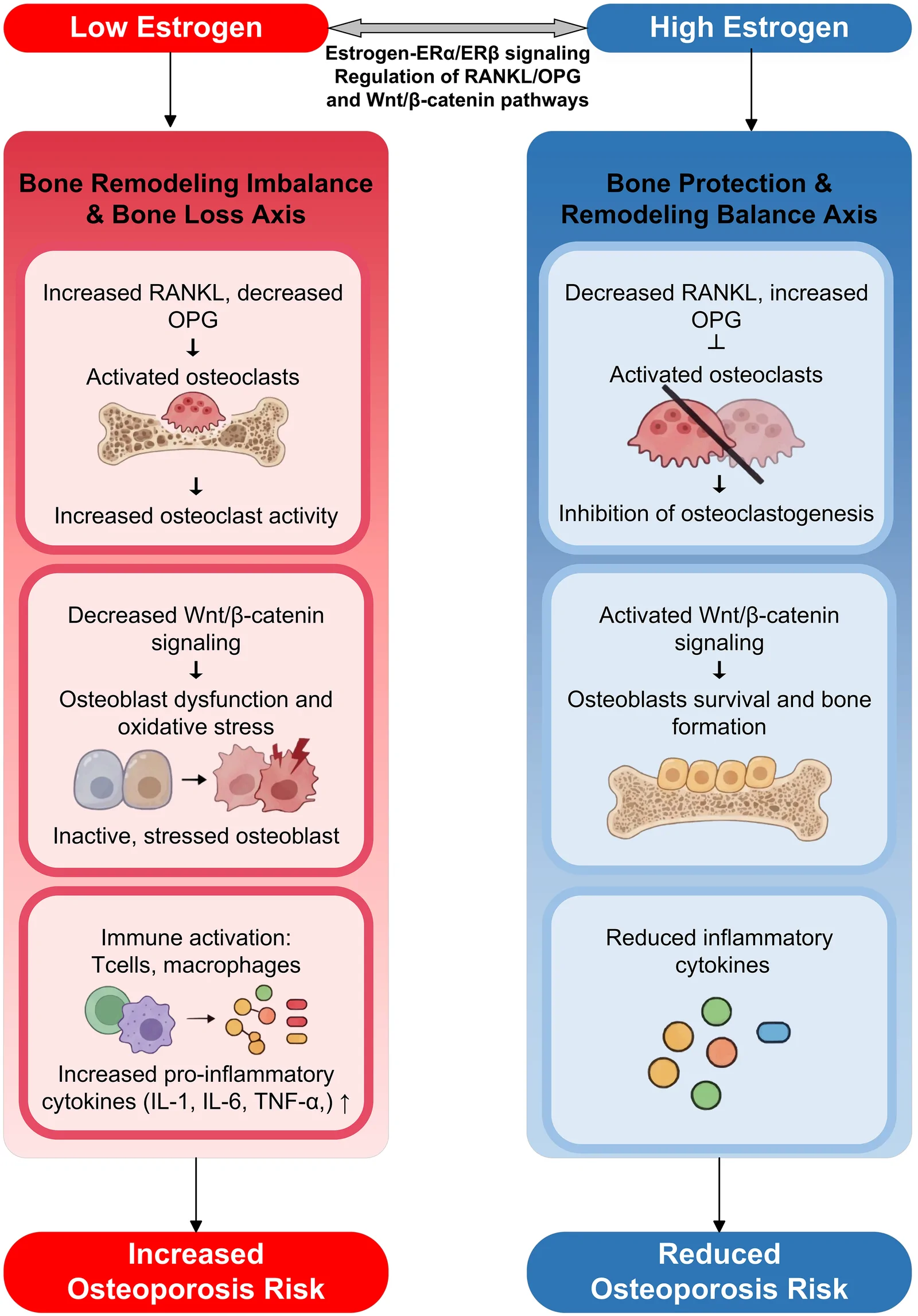
Bidirectional effects of estrogen on osteoporosis and bone remodeling in females. This schematic illustration explains the bidirectional effects of estrogen on regulating bone homeostasis through ERα/ERβ-mediated signaling pathways. Under low estrogen conditions (left), disruption of the RANKL/OPG balance (increased RANKL and decreased OPG) promotes osteoclast differentiation and activity, leading to increased bone resorption. Concurrently, suppression of Wnt/β-catenin signaling impairs osteoblast function, enhances oxidative stress, and reduces bone formation. Estrogen deficiency also activates immune cells (T cells and macrophages), increasing pro-inflammatory cytokines (IL-1, IL-6, TNF-α), which further exacerbate osteoclastogenesis and bone loss, ultimately increasing osteoporosis risk. In contrast, high estrogen levels (right) restore bone remodeling balance by decreasing RANKL and increasing OPG, thereby inhibiting osteoclastogenesis. Activation of Wnt/β-catenin signaling promotes osteoblast survival and bone formation, while reduced inflammatory cytokine production contributes to skeletal protection, resulting in decreased osteoporosis risk. ERα, estrogen receptor alpha; ERβ, estrogen receptor beta; RANKL, receptor activator of nuclear factor-κB ligand; OPG, osteoprotegerin; IL-1, interleukin 1; IL-6, interleukin 6; TNF-α, tumor necrosis factor-alpha; Wnt, Wingless/integrated signaling pathway. Created by the authors based on Refs (126, 145, 146).

Estrogen is important for skeletal homeostasis. It exerts its effects by regulating bone remodeling, modulating of osteoblast and osteoclast, and inhibiting pro-inflammatory signaling (126). Estrogen mainly acts through ERα and ERβ, which are expressed in osteoblasts, osteoclasts, and osteocytes, in which ERα is the dominant mediator of estrogen’s osteoprotective effects (145). Upon binding to ERα, estrogen initiates genomic signaling to regulate the receptor activator of nuclear factor-κB ligand (RANKL)/osteoprotegerin (OPG) system and the Wnt/β-catenin pathway regulation of osteoclast genesis to inhibit osteoclast genesis, promotion of osteoblast survival, and maintenance of balanced bone (146). Through ERs and their downstream canonical and non-canonical pathways, estrogen plays an indispensable role in maintaining bone mass, bone microstructure, and skeletal integrity (126). The mechanisms by which estrogen regulates osteoclastogenesis, osteoblast survival, and bone remodeling are summarized in **Figure 7**.

Estrogen deficiency increases the production of RANKL while reducing the expression of OPG, thereby enhancing the differentiation and activity of osteoclasts (146). Estrogen deficiency also functionally inhibits Wnt/β-catenin signaling, which causes impaired osteoblast differentiation and reduced bone formation, further exacerbated by increased oxidative stress and dysfunction of osteoblasts (147). Additionally, estrogen deficiency stimulates activated T cells and macrophages producing pro-inflammatory cytokines, such as IL-1, IL-6, and TNF-α, which further accelerates high-turnover bone loss (146). Simultaneously, the reduction of TGF-β and the increase of IL-7 also activate T cells (126). These cytokine changes exacerbate the RANKL/OPG imbalance that drives osteoclast activation (148). Estrogen deficiency has profound immunological consequences and promotes the pathogenesis of postmenopausal osteoporosis through osteoimmunological mechanisms (126). Therefore, the sharp decline of estrogen following menopause alters the remodeling balance, causing accelerated bone resorption, and leading to a gradual decrease in bone mineral density (126).

### 9.3 Clinical implications and hormone replacement therapy in osteoporosis

Given the accelerated bone loss following menopause, HRT remains one of the most effective interventions for preserving bone mass and preventing osteoporotic fractures (144). The use of HRT varies greatly depending on the type and timing of menopause. WHI trial demonstrated that estrogen plus progesterone therapy for healthy women with intact uterus, and estrogen alone therapy for women who have undergone hysterectomy, can increase bone area density (aBMD) and reduce the risk of fractures (149, 150). The protective effects on aBMD begin to manifest shortly after HRT but disappear after the cessation of HRT (151). The latest European Society of Endocrinology Guidelines published in 2025 for the management of menopause states that bone specific agents, not HRT, are considered the preferred treatment for postmenopausal women with diagnosed osteoporosis (152).

In addition, other factors, including early menopause, inadequate intake of calcium or vitamin D, lack of physical activity, low body mass index, smoking, excessive alcohol consumption, as well as personal or family history of fractures, also have been proven to cause bone loss (126).

## 10 Discussion

Menopause is a fundamental physiological process of systemic endocrine reprogramming, characterized by a sharp decline in ovarian estrogen and progesterone levels, a relative dominance of androgens, and compensatory changes in gonadotropins and local steroid metabolism. This transformation reshapes immune function, vascular physiology, metabolic status, brain aging, bone remodeling, and the tumor microenvironment, thereby fundamentally altering the risk trajectory of metabolic cardiovascular diseases, neurodegenerative diseases, osteoporosis, and hormone-sensitive cancers in the human body. Despite significant in-depth understanding in mechanism research, critical knowledge gaps still constrain the translation of these research achievements into precise, mechanism-based intervention measures.

Despite significant progress has been made, several challenges remain. The potential benefits and risks of HRT across different disease contexts are summarized in **Table 1**. Clinically, the field is transitioning toward a precision medicine framework that synergizes conventional risk factors with high-resolution molecular signatures of sex steroid signaling. This integration encompasses not only estrogen receptor 1 (ESR1)/estrogen receptor 2 (ESR2) genetic variants and estrogen-responsive transcriptomic signatures but also the “estrobolome”—the collective enteric microbial genome responsible for estrogen metabolism—alongside associated lipidomic and coagulation profiles. Such multi-dimensional biotyping will enable more granular patient stratification, facilitating personalized interventions that account for systemic hormonal, metabolic, and microbial landscapes. A central focus is the microbiome-gut axis, specifically the regulatory role of microbial β-glucuronidase in modulating the enterohepatic circulation of estrogens. Addressing these knowledge gaps necessitates longitudinal, multi-omic studies within well-phenotyped cohorts, leveraging high-resolution spatial mapping to uncover the intricate cross-talk between the gut, immune system, adipose tissue, and central nervous system.

**Table 1:** Comparison of risks and benefits of hormone replacement therapy for various diseases.

Diseases	Benefit	Risk
CVD	• Reduces the incidence of CVD in postmenopausal women. • Reduces vasoconstriction and stress-related blood pressure increases. • Reduces all-cause mortality or CHD when started at the optimal time (under age 60 and at or near menopause). • Beneficial effects on healthy endothelium.	• Little benefit in primary or secondary prevention. • Increase the risk of stroke and venous thromboembolic events. • Harmful effects in the presence of established atherosclerotic plaques (Healthy Endothelium Hypothesis). • Combined estradiol and testosterone treatment is associated with a higher risk of severe aortic calcification.
SLE	• Effective for managing menopausal symptoms in stable patients. • No significant increase in disease activity overall.	• Increase in the incidence of mild-to-moderate flares • Increased risk of venous thrombosis and stroke.
RA	• Protective effect in RA patients dominated by T cells. • Reduce the risk of RA. • Reduce the disease activity of RA • Significant positive effect on bone density.	• Increase the risk of disease deterioration for patients with seropositive RA exhibited obvious B-cell activation features • Contradictory evidence: Some studies show no effect on RA incidence.
Dementia	• Alleviates depressive symptoms and cognitive decline. • Reduce risk of dementia and possible excess risk of dementia	• Initiation after age 65 or long after menopause may increase the risk of dementia. • Possible increased risk in women with pre-existing cerebrovascular disease. • Progestin may antagonize the protective effects of estradiol.
Tumor	• Partially reverses the effects of ageing on immunity. • Estrogen combined with a natural progesterone and estrogen supplementation alone decrease breast cancer risk. • Combined estrogen–progestin is associated with reduced CRC incidence. • Reduced risk of conventional adenomas.	• Combination of estrogen and a progestogen may increase cancer risks in hormone-responsive tissues.
Osteoporosis	• Significant bone-protective effects in postmenopausal women. • Increase aBMD. • Reduce the risk of fractures. • Restore bone remodeling balance by inhibiting osteoclast activity and supporting osteoblast survival. • Effects on aBMD appear shortly after initiation. • Estrogen combined with progesterone has an additive beneficial effect on bone health.	• Protective effects on aBMD disappear after cessation of HRT. • Not the preferred treatment for women with diagnosed osteoporosis. • Effectiveness varies depending on type and timing of menopause.

CHD, coronary heart disease; CRC, colorectal cancer; CVD, cardiovascular disease; HRT, hormone replacement therapy; RA, rheumatoid arthritis; SLE, systemic lupus erythematosus; aBMD, bone area density.

## 11 Conclusion

In conclusion, current evidence supports a view of menopause as a systemic and dynamic endocrine remodeling process. The interplay between abrupt ovarian hormone withdrawal, compensatory intracrine steroidogenesis, immune and stromal reprogramming, and microbiome signals collectively determines postmenopausal disease risk across organ systems. Advances in genomics, single-cell and spatial technologies, systems epidemiology, and computational modeling now provide the methodological framework to link molecular perturbations with clinical phenotypes in a temporally and tissue-resolved manner.

Bridging fundamental mechanisms with clinical applications will require integrative, leveraging systems biology and precision medicine approaches. By strategically targeting both the upstream hormonal and the downstream effector pathways in metabolism, immunity, vasculature, and stroma, it may be possible to design innovative, personalized strategies to prevent and treat cardiometabolic disease, neurodegeneration, osteoporosis, and hormone-sensitive cancers in postmenopausal women. It may be possible to develop innovative strategies for women’s health across the lifespan and other conditions linked to menopause.
